# Using Theory to Conduct Qualitative Research with People Aging with Disability

**DOI:** 10.1093/geroni/igaf122.2497

**Published:** 2025-12-31

**Authors:** Tracie Harrison

**Affiliations:** University of Iowa College of Nursing, Iowa City, Iowa, United States

## Abstract

The use of theory to shape analysis of qualitative data while planning the study, collecting data in the field, and analyzing the data through select methods is necessary to arrive at meaningful findings in response to a research question. The lack of theoretical guidance in gerontological research within a biased interpretation of meaning and/or historical information can result in skewed findings. Although books and articles have been written about the process of using theory in qualitative research few have clarified how the study of aging populations can challenge disciplinary paradigms resulting in ethical concerns. This can leave the interpretation of complex data to a re-imagination or a best-alignment type of conclusion. In this examination of process and outcome between theory and data, the author suggests that an awareness of paradigmatic conflict must be identified or the voice of people from disparate backgrounds who have lived through multiple transitions with age will be lost, reinforcing disciplinary or analyst beliefs while disregarding complex dissention. Using data from ethnographic data collected from 133 people (age 55 and older) with 3-4 community interviews each, I demonstrate how conflict threaded along historical, individual, and disciplinary paradigms may exist. Further, the use of theory to anticipate the layering of ethical conflict within the interpretation of findings is provided.

